# Ethnic Differences in Preferences for Lifestyle Intervention among Women after Childbirth: A Multi-Methods Study in Australia

**DOI:** 10.3390/nu15020472

**Published:** 2023-01-16

**Authors:** Mingling Chen, Maureen Makama, Helen Skouteris, Lisa J. Moran, Cheryce L. Harrison, Tammie Choi, Siew Lim

**Affiliations:** 1 Monash Centre for Health Research and Implementation, School of Public Health and Preventive Medicine, Monash University, Clayton, VIC 3168, Australia; 2 Health and Social Care Unit, School of Public Health and Preventive Medicine, Monash University, Melbourne, VIC 3004, Australia; 3 Warwick Business School, University of Warwick, Coventry CV4 7AL, UK; 4 Department of Nutrition, Dietetics and Food, School of Clinical Sciences, Monash University, Notting Hill, VIC 3168, Australia; 5 Eastern Health Clinical School, Monash University, Box Hill, VIC 3128, Australia

**Keywords:** ethnicity, lifestyle intervention, postpartum, qualitative, survey

## Abstract

Postpartum weight retention contributes to maternal obesity and varies by ethnicity. Despite the well-established benefits of lifestyle intervention on weight management, little is known about how to engage postpartum women effectively, especially among ethnic minority groups. This multi-methods study aimed to explore ethnic differences in women’s preferences for lifestyle intervention after childbirth. Women within five years of childbirth and living with their youngest child in Australia were recruited in an online survey (*n* = 504) and semi-structured interviews (*n* = 17). The survey and interview questions were structured based on the Template for Intervention Description and Replication (TIDieR) framework. Ethnic groups were categorized as Oceanian, Asian and Other according to the Australian Bureau of Statistics. Chi-square tests were used to compare the preferred intervention characteristics between groups. Qualitative data were thematically analysed. The survey showed that most women across all ethnic groups were interested in receiving lifestyle support in the early postpartum period (from 7 weeks to 3 months postpartum). All ethnic groups preferred a regular lifestyle intervention delivered by health professionals that promotes accountability and provides practical strategies. However, Asian women had a higher desire for infant care and a lower desire for mental health in the intervention content compared with Oceanian women. Moreover, Asian women were more likely to favour interventions that are initiated in a later postpartum period, over a shorter duration, and with less intervention frequency, compared with Oceanian women. The interviews further indicated the need for intervention adaptations in the Asian group to address the cultural relevance of food and postpartum practices. These ethnic-specific preferences should be considered in the development of culturally appropriate intervention strategies to optimize engagement in healthy lifestyles among the targeted ethnic groups.

## 1. Introduction

Obesity is increasing rapidly in most population groups around the world [1]. Postpartum weight retention is a significant contributor to the development of obesity in women of childbearing age [2]. The inability to return to pre-pregnancy weight after excess gestational weight gain or additional weight gain postpartum increases the risk of long-term maternal obesity, cardiovascular disease, metabolic syndrome, as well as adverse neonatal and obstetric outcomes in subsequent pregnancies [3,4,5]. Importantly, postpartum weight retention has been found to vary by ethnicity [6]. Studies in Europe and the US showed that ethnic minority women with a non-European origin, such as Asian, Middle Eastern and African groups, experience higher weight retention postpartum compared to women of European origin [7,8,9]. The risk of type 2 diabetes and cardiovascular disease is also higher among ethnic minority women, particularly those from Asian and African backgrounds, as reported in Australia, Europe and the US [10,11,12]. These health disparities by ethnicity may be attributable to differences in physiological, behavioural, environmental, social and cultural factors among ethnic groups [6,10,13].

The postpartum period provides an important window of opportunity to target lifestyle behaviour changes and weight management to reduce long-term obesity-related risks [6]. However, low participation and poor engagement remain significant challenges in lifestyle interventions among postpartum women [6,14]. The main barriers to maintaining a healthy lifestyle postpartum are unique to this life stage, such as fatigue, time constraints, childcare needs and competing priorities [15]. Moreover, women from ethnic minority groups may have additional barriers associated with cultural beliefs and traditions that could impede their effective engagement in healthy diets, exercise and weight management postpartum [16,17,18]. These may include food taboos and physical activity restrictions after childbirth in Asian cultures [19,20], traditional clothing practices and conservative gender norms in Middle Eastern cultures [21,22] and appreciation of larger body sizes in African cultures [16,23]. These differential needs may necessitate the cultural adaptation of postpartum lifestyle interventions for different ethnic groups to enhance engagement and intervention effectiveness [24]. Adapting an intervention to achieve a cultural fit involves adaptations in the intervention content and process [25]. Content adaptation makes the messages culturally relevant and acceptable to the target population, while process adaptation ensures the delivery approaches are appropriate for the participants to optimize reach and participation [25]. There is currently a paucity of evidence to inform both content and process adaptations in postpartum lifestyle interventions for specific ethnic groups. Understanding the preferences for intervention content and delivery approaches of the target ethnic groups based on formative research is essential to guiding cultural adaptation and thus the successful implementation of interventions in the populations [24].

Australia is a multicultural society with almost 30% of its population born overseas [26]. The largest overseas-born population includes those born in Asia, comprising Southern (e.g., Indian, Sri Lankan), Northeast (e.g., Chinese, Korean) and Southeast (e.g., Filipino, Vietnamese) Asian groups [27]. Although Asian and other ethnic minority groups are at a high risk of postpartum weight retention and obesity-related diseases in later life, little is known about the optimal intervention strategies to engage these populations in postpartum lifestyle interventions. Previous Australian studies explored the lived experiences and factors influencing lifestyle behaviours in women after childbirth from Indian [28], Chinese [29,30], Middle Eastern [30], and other ethnic minority backgrounds [18,31], suggesting the need for culturally sensitive and appropriate postnatal service to address barriers in language, food practices and cultural role expectations, as well as provide adequate social support. However, no studies have systematically examined the intervention preferences of postpartum women from different ethnic groups, both in terms of intervention content and delivery approaches.

Therefore, the aim of this study was to explore and compare preferences for the content and delivery methods of lifestyle intervention after childbirth among women from different ethnic backgrounds living in Australia to inform the development of culturally appropriate interventions.

## 2. Materials and Methods

This is a multi-methods study involving a quantitative survey and qualitative interviews. The survey was performed to provide a broader overview of women’s preferences for lifestyle interventions in a larger sample, while semi-structured interviews were conducted to gather deeper insights into women’s needs and perspectives. The use of multiple methods provided a rich understanding of women’s perspectives with complementary strengths in both approaches. Eligible participants in both survey and interviews were women who were aged 18 years or above, had given birth within the past five years, were living with their youngest child in Australia and were not currently pregnant. This study was approved by the Monash University Human Research Ethics Committee (project number: 29273) and Monash Health Human Research Ethics Committee (reference number: RES-19-0000-685A). Survey participants provided online consent while interview participants provided audio-recorded verbal consent.

### 2.1. Survey

#### 2.1.1. Participant Recruitment

A nationwide cross-sectional survey was conducted online in November 2021. The questionnaire was developed on an online survey platform (Qualtrics, Provo, UT, USA) [32]. Women were recruited through an external cross-panel market research provider with a well-established database of over 200,000 Australian members [33]. The study participants were broadly representative of the Australian demographic populations according to the Australian Bureau of Statistics (ABS) by age, sex and location of residence (state/territory) [34]. Participants were screened for eligibility before being directed to the survey.

#### 2.1.2. Data Collection

The survey (Supplementary File S1) was designed by the research team to understand the preferences for lifestyle interventions in women after childbirth [35], as well as factors affecting their engagement with diet [36] and physical activity [37], including psychological distress [38], sleep patterns [39], parenting roles [40], and risk perception for type 2 diabetes [41] and cardiovascular disease [42]. The current study focused on ethnic differences in women’s preferences for lifestyle interventions after childbirth.

Questions regarding preferences for intervention content and delivery approaches were designed based on the Template for Intervention Description and Replication (TIDieR) framework [35], which is a standard publication checklist to systematically describe intervention characteristics, including What (intervention content), Who (intervention provider), How (delivery mode), Where (location of intervention), When and How much (intervention commencement time, duration and frequency). For intervention content, participants were asked to indicate what information they would like to be included in lifestyle interventions after childbirth with the option to select multiple responses on health knowledge (e.g., women’s health, children’s health, mum’s diet, exercise after birth) and skills and strategies for lifestyle management (e.g., how to set aside time for health, someone to monitor my progress, send me reminders and prompts). For delivery approaches, participants were asked to select their preferred intervention provider, delivery mode, location of intervention, intervention commencement time, duration and frequency by using single or multiple-choice questions, as shown in Supplementary File S1. Women’s interest (yes or no) in participating in lifestyle interventions after childbirth was also assessed.

Demographic characteristics were collected, including participants’ age, age of their youngest child, number of children, ethnicity, country of birth, years lived in Australia, marital status, education, employment and household income. Ethnicity was defined by self-reported ethnic or cultural background according to the classification by the ABS [43]. As people from Oceanian and Asian backgrounds represent the two largest demographic populations in Australia [26], we categorized ethnicity into three broad ethnic groups for analysis: Oceanian (Australian, New Zealander, Aboriginal and Torres Strait Islander, Maori and Pacific Islander), Asian (Northeast Asian, Southeast Asian, Southern and Central Asian), and Other (Northwest European, Southern and Eastern European, North African and Middle Eastern, North American, South American, Central American, Caribbean Islander, Central and West African, Southern and East African). Despite the fact that Indigenous Peoples, including Australian Aboriginal, Torres Strait Islanders, Maori and Pacific Islanders, were included in the Oceanian group as per the classification by the ABS, we acknowledge their unique cultural identities and have conducted sensitivity analysis on this group separately.

The questionnaire was available in English and Chinese to facilitate participants’ engagement using their preferred languages, as the most commonly spoken language other than English in Australia is Chinese (i.e., Mandarin and Cantonese) and Chinese migrants tend to have poor English proficiency [44]. Although Indians have outnumbered Chinese migrants as the largest Asian migrant population in Australia according to the 2021 Census [26], Indian migrants are generally proficient in English [44]. The survey was pilot tested within a small sample (*n* = 4, data not included in analysis). Minor modifications were made from the pilot test to improve the clarity of questions and to optimize the survey flow.

#### 2.1.3. Data Analysis

Demographic characteristics and preferred intervention characteristics were presented as mean ± standard deviation (continuous variables) or percentages (categorical variables) by ethnic groups. Chi-square tests were used for categorical variables to examine differences in intervention preferences between groups, with significance set at an α level of *p* < 0.05. Bonferroni correction was applied for multiple comparisons. Multivariable logistic regression analyses were conducted to examine the association between ethnicity and preferred intervention characteristics that were significantly different between ethnic groups on Chi-square tests. The models were adjusted for a priori determined potential confounders, including age, education, employment, household income and number of children. Odds ratios and 95% confidence intervals were reported for each ethnic group. Considering Indigenous women have different cultural barriers and health needs [45] compared with the other Oceanian women (i.e., Australian and New Zealander), sensitivity analysis was conducted by excluding the data on Indigenous women to test the robustness of the main results, while presenting the data on this group separately. Statistical analysis was performed using SAS 9.4 (SAS Institute Inc., Cary, NC, USA).

### 2.2. Semi-Structured Interviews

#### 2.2.1. Participant Recruitment

Semi-structured in-depth interviews were conducted between May 2020 and July 2020 as well as in May 2022, with the aim of gaining insights into the intervention preferences of women after childbirth. To focus on the two largest broad cultural groups in Australia, purposive sampling was used to recruit women from Oceanian and Asian backgrounds (as described above) through researchers’ networks, word of mouth, snowballing technique, and the Cardiometabolic Health Implementation Research in Postpartum (CHIRP) individual consumer group [46]. Interviews were conducted via video-conference (Zoom software, Zoom Video Communications Inc., San Jose, CA, USA) to facilitate accessibility and participation. A digital gift card was given to each participant as a reimbursement.

#### 2.2.2. Data Collection

Interviews were conducted by a female accredited practicing dietitian (S.L. or L.J.M.) or doctoral researcher (M.C.) in lifestyle interventions for postpartum women who were fluent in English, Mandarin and Cantonese. Although English and Chinese languages were offered for the interviews, all participants opted to complete the interviews in English. Some participants had a prior relationship with the interviewers and were aware of the interviewers’ research interests due to the nature of convenience sampling. Each interview lasted 30 to 50 min. The interview guide (Supplementary File S2) included general orientation questions followed by open-ended questions relating to women’s perspectives of healthy lifestyle, facilitators and barriers to lifestyle management, preferences for lifestyle interventions after childbirth, and perspectives on cost of the lifestyle program. Questions on intervention preferences were developed based on the TIDieR framework [35]. The interview guide was pilot tested in two women after childbirth (data not included in analysis). Interview participants also completed a short online questionnaire which collected demographic data, including age, ethnicity, country of birth, years lived in Australia, last date to give birth, number of children, marital status, education, employment and household income. None of the participants were from an Indigenous background.

#### 2.2.3. Data Analysis

Interviews were audio-recorded and transcribed verbatim using a professional transcription service. The transcripts were sent to all participants for verification and five of them (Oceanian *n* = 2, Asian *n* = 3) responded with no changes suggested. Qualitative data were coded through an open coding approach using NVivo 12 (QSR International Pty Ltd.). One researcher (M.C. or M.S.) independently coded all the transcripts and a second researcher (S.L. or L.J.M.) coded a subsample (at least 10%) until a consensus on the coding was reached between the coders. Any discrepancy was resolved by discussion. Thematic analysis was performed based on the inductive codes which were clustered into subthemes and main themes according to the Braun and Clarke method [47]. Themes were developed and reviewed by two researchers together (S.L. and M.C.). Data saturation was reached within the Oceanian and Asian groups respectively. Quantitative data on demographic characteristics were descriptively analysed using SAS 9.4 (SAS Institute Inc., Cary, NC, USA). The qualitative research was conducted and reported according to the Consolidated Criteria for Reporting Qualitative Research (COREQ) guidelines [48].

## 3. Results

### 3.1. Survey

Out of 874 women screened, 577 were eligible and consented to participate. For the current study, we further excluded women if they had missing data on the TIDieR elements (*n* = 64) or ethnicity (*n* = 9). In total, 504 women were included in the analysis with the following ethnic groups: 52.6% Oceanian (*n* = 265), 34.5% Asian (*n* = 174) and 12.9% Other (*n* = 65). Most participants (99.4%) completed the survey in English. The demographic characteristics of participants are presented in Table 1 and Table S1. The survey responses on the intervention preferences are presented in Table 2. The majority of women across all ethnic groups (Oceanian 90.2%, Asian 93.7%, Other 86.2%) showed interest in participating in a lifestyle intervention after childbirth.

#### 3.1.1. What (Intervention Content)

For health knowledge, the most preferred information to be included in the intervention was women’s health, regardless of ethnic groups. The top three most preferred information in the Oceanian and Other ethnic groups also included women’s mental health and exercise after birth, while in the Asian group it included women’s weight management, infant care and children’s health, with infant care and children’s health ranking equally. Significant differences between the three ethnic groups were found in the preferences for infant care (*p* = 0.024) and mental health (*p* = 0.006). Multiple comparisons showed that Asian women had a higher desire for information on infant care (71.3% vs. 59.6%, *p* = 0.038) and a lower desire for information on their mental health (69.0% vs. 81.5%, *p* = 0.007) compared to Oceanian women.

In terms of skills and strategies, the most preferred options (by around 60% of the participants regardless of ethnic groups) were social support for health and someone to monitor their progress. Significant differences between the groups were found in the preferences for receiving intervention reminders and prompts (*p* = 0.034); Asian women were more likely to prefer intervention reminders and prompts compared to women from the Other ethnic group (63.2% vs. 44.6%, *p* = 0.028).

#### 3.1.2. Who (Intervention Provider)

The most preferred intervention provider for all ethnic groups was health professionals with expertise in women’s health as compared with another mum or other providers. Significant differences were observed between the groups (*p* = 0.035). Multiple comparisons revealed that women from the Other ethnic group were more likely to favour health professionals with expertise in women’s health compared to the Asian group (98.5% vs. 87.9%, *p* = 0.037).

#### 3.1.3. When and How Much (Intervention Commencement Time, Duration and Frequency)

Within each ethnic group, most women preferred interventions commencing at 7 weeks to 3 months postpartum instead of the later months after childbirth, and an intervention duration lasting one year instead of a shorter duration of 6 months or less. The most preferred intervention frequency in Oceanian and Asian women was monthly, while that in women from the Other ethnic group was fortnightly. For each session of the intervention, most women from the Asian and Other ethnic groups preferred a shorter duration (between 15 and 30 min), while most Oceanian women preferred a slightly longer duration (between 30 and 45 min). Significant differences between the groups were seen in all these intervention preferences (all *p* < 0.05). Multiple comparisons showed that Asian women were more likely to favour interventions that are initiated later following birth (*p* = 0.002), over a shorter duration (*p* = 0.005), and with shorter sessions each time (*p* = 0.008) than Oceanian women. Asian women also tended to favour less frequent interventions than the Oceanian and Other ethnic groups (both *p* < 0.001).

#### 3.1.4. How (Delivery Mode)

The most preferred delivery format was individual consultation for all ethnic groups. However, women from the Oceanian and Other ethnic groups preferred the individual consultation to be delivered face-to-face, while Asian women had no preferential modes in regards to face-to-face or technology-mediated delivery of individual sessions. Significant between-group differences were found in the preferences for technology-mediated individual (*p* = 0.026) and group consultations (*p* = 0.021). After multiple comparisons, there were no significant ethnic differences in individual video or phone consultations, but a significantly higher preference for group video consultations in Asian women compared to Oceanian women (29.9% vs. 19.6%, *p* = 0.040).

#### 3.1.5. Where (Location of Intervention)

The most preferred location of intervention was during maternal and child health (MCH) nurse visits for all ethnic groups. The MCH service is a free universal primary health service available for all families with children from birth to school age in Australia [49]. No ethnic differences were found in the preferences for the location of intervention.

#### 3.1.6. Multivariable Regression Analysis

Table S2 shows the multivariable regression results. After adjusting for age, education, employment and household income (Model 1), significant differences remained between Oceanian and Asian women in the preferences for intervention content on infant care and mental health, intervention commencement time, duration and frequency, as well as technology-mediated individual and group consultations. When additionally adjusting for number of children (Model 2), there were no longer ethnic differences in the preferences for intervention content, but differences remained for intervention commencement time, duration, frequency and delivery mode between Oceanian and Asian women. Both two models revealed no significant differences between the Oceanian and Other ethnic groups.

#### 3.1.7. Sensitivity Analysis

The sensitivity analysis by excluding the data on Indigenous participants (*n* = 27) showed no significant ethnic differences in group video consultations after multiple comparisons. No other results were substantially changed (Table S3). The survey responses for the intervention preferences in Indigenous participants are presented separately in Table S4.

### 3.2. Semi-Structured Interviews

Eighteen eligible women expressed interest with one lost to contact prior to the interview. In total, 17 women (Oceanian *n* = 8, Asian *n* = 9) completed the interviews. The demographic characteristics of the participants are shown in Table 1 and Table S1. The qualitative analysis identified four main themes: (1) Practical and tailored strategies including cultural adaptations; (2) Early and regular support, with considerations of cultural postpartum practices; (3) Accessible delivery by health professionals; and (4) Building a strong support network for health. Table 3 shows the subthemes and themes mapped to the TIDieR framework with illustrative quotations.

#### 3.2.1. Theme 1: Practical and Tailored Strategies including Cultural Adaptations

Women in both ethnic groups suggested interventions should be tailored according to individual needs, with consideration of specific health conditions, including diastasis recti or gestational diabetes, and social conditions such as being a single parent. Asian women, in particular, expressed the need for cultural adaptations such as the inclusion of traditional recipes to optimize health. Both ethnic groups reported they do not need further health information due to experiencing overload from the information received. Instead, they expressed a strong desire for practical strategies and skills that could help them to better manage time and incorporate healthy behaviours into their busy routines. Women also stated that accountability plays an important role in maintaining a healthy lifestyle. Interventions that make them accountable and help them stick to health plans, for example, through regular check-ins, reminders and monitoring, were favoured.

#### 3.2.2. Theme 2: Early and Regular Support, with Considerations of Cultural Postpartum Practices

Both ethnic groups generally supported lifestyle interventions that commence early, with preferences ranging from 6 weeks to 6 months following birth. Commencement earlier than these times was not preferred due to barriers with the care of a newborn, adapting to motherhood and recuperating from childbirth. Asian women additionally expressed concerns with cultural postpartum practices that could impede their early engagement in lifestyle intervention. Traditionally Asian women follow postpartum confinement (30 to 40 days after birth) during which period some restrictions are advised, including staying indoors and limiting activity.

In terms of intervention frequency, both ethnic groups preferred regular support on a weekly, fortnightly or monthly basis. They thought regular support would keep them motivated in maintaining a healthy lifestyle, with the frequency depending on their time availability or the complexity of the content received. For intervention duration, women had different preferences ranging from weeks and months to one year. The rationale for the preferred duration focused on the time needed to develop a routine or to form a healthy habit around lifestyle changes. In addition, several participants preferred a short-term intervention due to concerns about diminished interest over time. For each session of intervention, women in both ethnic groups favoured a short duration (maximum 30–60 min), as a longer duration might pose a challenge for infant care.

#### 3.2.3. Theme 3: Accessible Delivery by Health Professionals

Both ethnic groups unanimously suggested MCH nurses as the ideal intervention provider due to the regular, integrated and credible service they provided in early postpartum. Participants also indicated the need for a range of allied health (dietitians, physiotherapists and psychologists) and fitness professionals (personal trainers, yoga and meditation instructors) to improve their physical and mental wellbeing. Conversely, general practitioners (GPs) were thought to be less helpful. Some women had negative experiences when seeking postpartum lifestyle advice from GPs, while others perceived the role of a GP as treating illness instead of providing general wellbeing support.

All the participants expressed that the lifestyle program should be accessible. This included interventions that can be incorporated into existing routine care, such as MCH visits and child immunisation appointments, and located in community settings (e.g., local library, gym or park). Online delivery was also preferred, with the advantages of easy accessibility, flexible scheduling and, in particular, reducing the risk of infection during the coronavirus disease 2019 (COVID-19) pandemic. Both ethnic groups expressed the desire for free or subsidized programs to uptake mothers and enhance engagement. The participants’ willingness to pay depended on their perceived value of the program, which was judged by intervention content, provider, timing and how the program would benefit them.

#### 3.2.4. Theme 4: Building a Strong Support Network for Health

Both ethnic groups stated that face-to-face delivery in a small group has significant benefits of social interaction and facilitates peer support. Understanding, encouragement and accountability from other mums who share similar experiences served as a great source of support during the transition into motherhood. Mother’s groups, for example, were considered a good opportunity to create such a support network, particularly for first-time mums. In addition, participants thought programs involving their babies and partners could address the need for childcare during the intervention, allowing them to balance self-care with parenting responsibilities and family commitments.

## 4. Discussion

This study provides important insights to inform the design of lifestyle intervention after childbirth for women from different ethnic backgrounds, especially for Oceanian and Asian groups in Australia. The findings suggest that women from all ethnic groups were interested in receiving lifestyle support in the early postpartum period. The survey and interviews found all ethnic groups preferred a regular lifestyle intervention delivered by health professionals which can promote accountability and provide practical strategies. However, several ethnic differences were identified from the survey. In terms of intervention content, Asian women had a higher desire for infant care but a lower desire for mental health compared to Oceanian women. Moreover, Asian women were more likely to favour interventions that are initiated later following birth, over a shorter duration and with less intervention frequency. The interviews further indicated the need for intervention adaptations in the Asian group to address culturally-relevant food and postpartum practices.

Our results showed Asian women were more likely to prefer knowledge on infant care than Oceanian women. The emphasis on infant care among Asian women could be due to traditional gender norms in parenting roles within Asian cultures [50]. East and Southeast Asian families are greatly influenced by Confucianism, which advocates the family over the individual, hierarchy over freedom and equality, and harmony over diversity and conflict [51]. Under this philosophical structure, fathers are considered as the head of the family and mothers are expected to take on childcare responsibilities and domestic matters. Likewise, South Asian families follow a patriarchal family structure in which fathers are typically responsible for financial support while mothers are the primary caregivers and nurturers to young children [50]. In addition, raising young children could be more challenging for Asian migrants due to lack of family support and unfamiliarity with health and community services in their new countries [52]. Hence, historical and cultural influences on parenting roles in addition to the environmental challenges associated with being a migrant may explain the higher desire for knowledge on infant care among the Asian women in our study.

On the other hand, Asian women tended to be less concerned with their mental health compared to Oceanian women. Previous studies have similarly found that Asian women have a low awareness of mental health or are reluctant to acknowledge mental health concerns, due to low mental health literacy or cultural barriers, such as fear of stigma and being considered as an unfit mother [28,53,54,55]. However, it is documented that Asian migrants are more vulnerable to poor mental health in the postpartum period as evident through higher rates of postpartum depression compared with women born in the host countries [53,56,57]. Social isolation, poor partner support and lower socioeconomic status are the main contributors to postpartum depression in Asian migrant women [53,56,57]. The mismatch between the high prevalence of postpartum mental disorders and the relatively low mental health demand in Asian women highlights the need to improve mental health awareness and build culturally appropriate support in this vulnerable group.

In terms of the delivery of intervention, while all ethnic groups endorsed regular support with a short session each time to accommodate their childcare needs, the Asian group preferred a lower-intensity intervention with less frequency and a shorter duration. Asian women’s preferences for low-intensity interventions may be explained by having less opportunity and time to focus on their own health management due to traditional gender norms in household responsibilities and cultural expectations of the role of mothers [28,50]. In addition, previous qualitative studies found that, in contrary to Westerners, some Asian subgroups, such as East Asian populations, prefer to receive health education in a top-down and didactic delivery manner rather than in an active participatory and explorative way [58,59]. Such a prescriptive approach may lead to interventions of shorter duration or less frequency. Low-intensity lifestyle interventions among Asian groups have been shown to successfully promote weight loss in the general population [60] and in postpartum women [61,62]. A recent systematic review [63] found the lowest intensity of intervention to induce significant weight loss postpartum was two in-person sessions with the provision of a home-based game console in Japanese women [62]. More studies are required to determine the optimal intensity of interventions (e.g., frequency, duration, number of sessions) in Asian postpartum women.

In addition, interventions for the Asian group should be culturally sensitive by considering their postpartum practices and food preferences. In most Asian cultures, traditional postpartum confinement practices are observed [20]. Postpartum confinement involves a series of practices, including dietary regulations such as high-calorie intake, limiting physical activity, bathing abstinence and traditional postpartum massage, which are believed to promote maternal recovery and the baby’s wellbeing [64]. Although postpartum practices vary across cultures, the time period commonly lasts one to a couple of months after childbirth [65]. Thus, the various cultural practices during the confinement period could greatly influence women’s engagement in lifestyle interventions in the early postpartum stage. A previous systematic review showed no clear pattern with the stage of intervention commencement (within 1–2 days to 3–12 months postpartum) and postpartum weight outcomes [66]. Aligning the intervention timing with women’s preferences may be more likely to increase participation and engagement. Like many other cultures, food holds significant meanings in Asian societies as it is deeply rooted within the social, economic and religious aspects of daily life [67]. Despite acculturation among Asian migrants in Western countries, the consumption of ethnic foods is a widespread practice among Asian groups as a way to maintain their ethnic identities [68]. In previous Australian studies, Asian groups frequently reported the dietary advice they received from health professionals was not culturally relevant, making it difficult for them to understand and follow [18,59,68]. Given the important role of healthy eating in weight management during and after pregnancy [63,69], there is a need for culturally appropriate dietary advice that can be understood, accepted and acted upon in Asian women.

Despite the differential preferences stated above, all ethnic groups also shared some common needs for lifestyle interventions. In line with previous studies [70,71], our results indicated the need for practical behavioural change strategies as well as accountability in postpartum women to accommodate competing priorities and make commitments to a healthy lifestyle during this challenging period. As women experience considerable changes in their physical, mental and social health following birth [72], a range of health professionals should be involved for their health support. One-on-one consultations can provide individualized feedback and home-based care for postpartum women [6,73], while small group delivery benefits from peer support which is important to alleviate emotional drain and encourage behavioural changes [74,75]. From our survey and interviews, combining the two formats in delivering an intervention may be beneficial for postpartum women. In light of the COVID-19 pandemic, online delivery was preferred due to the reduced risk of exposure in addition to its accessibility and flexibility. This seemed to be more evident in Asian women, who are more likely to perceive COVID-19 as a threat to their personal health than their white counterparts [76]. Furthermore, with the competing demands for childcare and household responsibilities, community-based lifestyle programs that encompass the child, mother and family may be effective in engaging postpartum women from all ethnic groups.

### Strengths and Limitations

To our knowledge, this is the first multi-methods study that has sought to explore and compare women’s preferences for lifestyle support after childbirth between ethnic groups. The use of a multi-methods approach resulted in complementary strengths by presenting the quantitative data of a larger sample of women with further explanations via qualitative interviews [77]. Moreover, by utilizing the TIDieR framework, the findings of this study can be used to aid in developing theory-informed interventions which are crucial to achieving success in changing behaviours [78]. With regard to the study limitations, we used a broad classification of ethnic groups as Oceanian, Asian and Other, which has masked the diverse cultural backgrounds within each of these categories. However, these broad recommendations improved our understanding on the two largest demographic populations in Australia. Due to the small sample size of Indigenous women in our study, we were unable to examine differences between this underserved group and other ethnic groups. The interview participants of the Asian group were English-speaking, highly educated, and had resided in Australia for more than 6 years. Thus, these findings may not be applicable to non-English-speaking new migrants. Furthermore, the ethnic differences revealed in our study may also be due to other factors, such as number of children, which should be taken into account in the development of interventions.

## 5. Conclusions

Our study revealed that women from all ethnic groups had a strong desire to receive lifestyle support after childbirth, but ethnic differences were found in their preferences for lifestyle interventions. These differences identified were not only in the intervention content but also in the delivery of the intervention, suggesting a need for both content and process adaptations when designing culturally appropriate interventions for postpartum women from different ethnic groups. Future research should consider these preferences and needs when developing and implementing postpartum lifestyle programs targeting individuals from different ethnic groups to optimize engagement and effectiveness.

## Figures and Tables

**Table 1:** Demographic characteristics of survey and interview participants.

Characteristics	Survey			Interview	
	Oceanian (*n* = 265)	Asian (*n* = 174)	Other (*n* = 65)	Oceanian (*n* = 8)	Asian (*n* = 9)
Age (years)	32.7 ± 5.9	34.3 ± 4.6	35.1 ± 5.2	36.4 ± 4.7	37.7 ± 4.8
Postpartum age (years)	2.1 ± 1.6	2.5 ± 1.6	2.7 ± 1.7	1.4 ± 0.3	2.1 ± 0.9
Number of children living in the household					
1	80 (30.2)	78 (44.8)	23 (35.4)	3 (37.5)	3 (33.3)
2	113 (42.6)	74 (42.5)	23 (35.4)	3 (37.5)	4 (44.4)
≥3	72 (27.2)	22 (12.6)	19 (29.2)	2 (25.0)	2 (22.2)
Born in Australia					
No	20 (7.6)	156 (89.7)	51 (78.5)	1 (12.5)	9 (100.0)
Years lived in Australia					
≤5 years	1 (0.4)	49 (28.2)	15 (23.1)	0 (0.0)	0 (0.0)
6 to 10 years	9 (3.4)	64 (36.8)	11 (16.9)	1 (12.5)	3 (33.3)
≥11 years	255 (96.2)	61 (35.1)	39 (60.0)	7 (87.5)	6 (66.7)
Marital status					
Never married	24 (9.1)	2 (1.2)	4 (6.2)	1 (12.5)	0 (0.0)
Married	143 (54.0)	161 (92.5)	44 (67.7)	5 (62.5)	9 (100.0)
De facto	78 (29.4)	7 (4.0)	12 (18.5)	1 (12.5)	0 (0.0)
Separated or divorced	19 (7.2)	2 (1.2)	5 (7.7)	1 (12.5)	0 (0.0)
Education					
High school	101 (38.1)	17 (9.8)	10 (15.4)	1 (12.5)	0 (0.0)
Diploma	61 (23.0)	21 (12.1)	15 (23.1)	0 (0.0)	0 (0.0)
Bachelor	58 (21.9)	70 (40.2)	24 (36.9)	1 (12.5)	2 (22.2)
Postgraduate	44 (16.6)	65 (37.4)	16 (24.6)	6 (75.0)	7 (77.8)
Employment					
Full-time	79 (29.8)	58 (33.3)	16 (24.6)	4 (50.0)	3 (33.3)
Part-time	101 (38.1)	60 (34.5)	28 (43.1)	4 (50.0)	5 (55.6)
Unemployed	82 (30.9)	54 (31.0)	20 (30.8)	0 (0.0)	1 (11.1)
Household income per year ($)					
≤49,999	48 (18.1)	19 (10.9)	10 (15.4)	0 (0.0)	1 (11.1)
50,000 to 99,999	70 (26.4)	62 (35.6)	19 (29.2)	0 (0.0)	0 (0.0)
≥100,000	136 (51.3)	79 (45.4)	33 (50.8)	6 (75.0)	5 (55.6)
Prefer not to answer	11 (4.2)	14 (8.1)	3 (4.6)	2 (25.0)	3 (33.3)

Data are presented as mean ± standard deviation or *n* (%).

**Table 2:** Survey responses to the preferences for lifestyle intervention after childbirth according to TIDieR framework.

TIDieR Elements	Oceanian (*n* = 265)	Asian (*n* = 174)	Other (*n* = 65)	*p* Value
Interest in lifestyle intervention				
Yes	239 (90.2)	163 (93.7)	56 (86.2)	0.170
Intervention content				
*Knowledge*				
Women’s health	222 (83.8)	148 (85.1)	55 (84.6)	0.934
Breastfeeding	184 (69.4)	115 (66.1)	45 (69.2)	0.750
Caring for my baby	158 (59.6)	124 (71.3)	37 (56.9)	0.024 ^a^
Children’s health	170 (64.2)	124 (71.3)	42 (64.6)	0.282
Mum’s diet	185 (69.8)	108 (62.1)	38 (58.5)	0.105
Exercise after birth	198 (74.7)	118 (67.8)	46 (70.8)	0.285
Weight management	187 (70.6)	132 (75.9)	42 (64.6)	0.196
Mental health	216 (81.5)	120 (69.0)	53 (81.5)	0.006 ^b^
Preventing diabetes or heart disease	82 (30.9)	67 (38.5)	17 (26.2)	0.118
*Skills and strategy*				
How to determine the credibility of health information	78 (29.4)	43 (24.7)	20 (30.8)	0.484
How to set goals and action plans for health	131 (49.4)	75 (43.1)	25 (38.5)	0.189
How to set aside time for health	136 (51.3)	83 (47.7)	35 (53.9)	0.636
Self-recording diet or physical activity	73 (27.6)	56 (32.2)	13 (20.0)	0.167
Monitoring blood tests and other health outcomes	65 (24.5)	45 (25.9)	18 (27.7)	0.858
Someone to monitor my progress	176 (66.4)	101 (58.1)	43 (66.2)	0.183
Send me reminders and prompts	151 (57.0)	110 (63.2)	29 (44.6)	0.034 ^c^
Social support for health	180 (67.9)	109 (62.6)	43 (66.2)	0.521
Questions to ask my doctor	114 (43.0)	76 (43.7)	23 (35.4)	0.481
Intervention provider				
Someone with expertise in women’s health, e.g., health professional	243 (91.7)	153 (87.9)	64 (98.5)	0.035 ^d^
Someone with expertise in children’s health, e.g., health professional	142 (53.6)	100 (57.5)	34 (52.3)	0.663
Another mum	72 (27.2)	45 (25.9)	15 (23.1)	0.792
Intervention commencement time				0.001 ^e^
6 weeks or earlier	93 (35.6)	52 (30.1)	24 (37.5)	
7 weeks to 3 months	112 (42.9)	65 (37.6)	31 (48.4)	
4 to 6 months	52 (19.9)	39 (22.5)	6 (9.4)	
After 6 months	4 (1.5)	17 (9.8)	3 (4.7)	
Intervention duration				0.014 ^f^
≤ 1 month	22 (8.5)	34 (19.7)	9 (13.9)	
3 months	34 (13.1)	29 (16.8)	9 (13.9)	
6 months	80 (30.8)	36 (20.8)	15 (23.1)	
1 year	124 (47.7)	74 (42.8)	32 (49.2)	
Intervention frequency				<0.001 ^g,h^
Every 6 months	13 (5.0)	11 (6.5)	3 (4.7)	
Every 3 months	35 (13.5)	46 (27.1)	10 (15.6)	
Every month	94 (36.3)	76 (44.7)	17 (26.6)	
Every fortnight	68 (26.3)	29 (17.1)	21 (32.8)	
Every week	49 (18.9)	8 (4.7)	13 (20.3)	
Duration of session				0.022 ^i^
Less than 15 min	15 (5.7)	10 (5.8)	5 (7.7)	
Between 15 and 30 min	97 (36.6)	94 (54.3)	27 (41.5)	
Between 30 and 45 min	110 (41.5)	48 (27.8)	23 (35.4)	
More than 45 min	43 (16.2)	21 (12.1)	10 (15.4)	
Delivery mode				
Individual video or phone consultation	101 (38.1)	86 (49.4)	22 (33.9)	0.026 ^j^
Individual face-to-face consultation	154 (58.1)	86 (49.4)	40 (61.5)	0.117
Group video consultation	52 (19.6)	52 (29.9)	11 (16.9)	0.021 ^k^
Group face-to-face consultation	98 (37.0)	64 (36.8)	28 (43.1)	0.631
Location of intervention				
Online	188 (70.9)	115 (66.1)	43 (66.2)	0.505
Maternal child health nurse visit	196 (74.0)	139 (79.9)	48 (73.9)	0.332
GP clinic	134 (50.6)	92 (52.9)	37 (56.9)	0.639
Mother’s group or playgroup	134 (50.6)	103 (59.2)	35 (53.9)	0.207

GP, general practitioner. Data are presented as *n* (%). ^a^ Significant pairwise differences between Oceanian and Asian (Bonferroni-adjusted *p* = 0.038). ^b^ Significant pairwise differences between Oceanian and Asian (Bonferroni-adjusted *p* = 0.007). ^c^ Significant pairwise differences between Asian and Other (Bonferroni-adjusted *p* = 0.028). ^d^ Significant pairwise differences between Asian and Other (Bonferroni-adjusted *p* = 0.037). ^e^ Significant pairwise differences between Oceanian and Asian (Bonferroni-adjusted *p* = 0.002). ^f^ Significant pairwise differences between Oceanian and Asian (Bonferroni-adjusted *p* = 0.005). ^g^ Significant pairwise differences between Oceanian and Asian (Bonferroni-adjusted *p* < 0.001). ^h^ Significant pairwise differences between Asian and Other (Bonferroni-adjusted *p* < 0.001). ^i^ Significant pairwise differences between Oceanian and Asian (Bonferroni-adjusted *p* = 0.008). ^j^ No significant pairwise differences found after Bonferroni correction. ^k^ Significant pairwise differences between Oceanian and Asian (Bonferroni-adjusted *p* = 0.040).

**Table 3:** Quotations from interview participants on the preferences for lifestyle intervention after childbirth according to TIDieR framework.

TIDieR Elements	Subthemes	Themes	Representative Quotes: Oceanian	Representative Quotes: Asian
What (intervention content)	No more information needed	Theme 1. Practical and tailored strategies including cultural adaptations	“I don’t really feel like I need more information. I feel like at the moment it’s information overload.”—O1 (Australian)	“What I don’t want is another booklet explaining how to be healthy. I think I’m just sick of booklets.”—A9 (Southern Asian)
	Accountability		“Look, ideal world for me, I think whatever the model is, it’s about having accountability to keep you on that track.”—O5 (Australian)	“The physio wrote me down on a daily to weekly basis, what type of exercise I should be doing. Gave me a demonstration, got me to practice, and then let me go off on my own and repeat them.”—A4 (Northeast Asian)
	Practical strategies		“…to think about how you can make protected pockets of time within your week to make sure that you do the things that are gonna help you, keep your energy and therefore your health and wellbeing up.”—O8 (New Zealander)	“On top of that, how we can incorporate exercise to busy lifestyle. It’s like setting goals and how to find time to do exercises.”—A8 (Southern Asian)
	Individually and culturally tailored		“There might be some sort of very tailored information in terms of how to incorporate those kinds of things when you do have kids. And particularly I guess it’s sort of, specific enough to be focused on single parents as well.”—O1 (Australian)	“I remember someone saying we eat a lot of dosa [traditional Indian food]. She said, ‘Okay, that’s fine. You just add this extra bit of lentil onto your dosa that makes it more protein-based rather than carb-based.’ I was like, ‘Oh, I didn’t know that.’ Just even traditional food is very carb-based and very unhealthy. If someone explained it to me as a mum, then it’s helpful for me.”—A9 (Southern Asian)
When and how much (intervention commencement time)	6 weeks to 6 months after childbirth	Theme 2. Early and regular support, with considerations of cultural postpartum practices	“In terms of exercising support, I think you don’t want to be encouraging people to be exercising too much in the first couple of months, just because I think you’re still recovering and getting used to everything.”—O7 (Australian)	“We have different traditions and customs. So you have to go through that one and a half months of postpartum… Once you have given birth, you just need to stay in the bed, most of the time cover yourself, cover the baby, cover yourself, cover the baby. Don’t go anywhere.”—A5 (Southern Asian)
When and how much (intervention duration)	Depends on needs		“I think maybe a short period would be better. I think long term, people lose interest.”—O4 (Australian)	“I think at least one year because once we start developing a routine and if we do that for a year, it’s gonna be a part of your lifestyle.”—A8 (Southern Asian)
When and how much (intervention frequency)	Weekly, fortnightly or monthly		“I think every few weeks or every month. I guess the other thing in those early days is the kind of haphazardness of your availability which is challenging.”—O8 (New Zealander)	“In terms of keeping a healthy lifestyle, if we don’t do it at least once a week, then it’s a bit hard to get back when we left it for too long. You kind of lost the motivation.”—A1 (Southeast Asian)
When and how much (duration of session)	Short session		“Keeping an eye on your babies, but up to an hour. An hour is a long time for a baby.”—O7 (Australian)	“Half an hour probably is good enough, because newborn they generally feed about like every two hours.”—A1 (Southeast Asian)
Who (intervention provider)	Maternal child health nurse	Theme 3. Accessible delivery by health professionals	“I liked my maternal child health nurse and my experience with that service. I did prioritize those appointments.”—O2 (Australian)	“I really enjoyed the maternal child health nurses. They were like everything in one.”—A7 (Southern Asian)
	Allied health and fitness professionals		“Allied Health, a personal trainer, you know, a physiologist who could set a program would be good.”—O4 (Australian)	“It’s that mental resilience for me personally. Physically, it’s those physio advise in relation to aches and pains that you get after birth or issues such as pelvic floor.”—A4 (Northeast Asian)
	Not GP		“For me a GP not so much, because I don’t really have any major health problems.”—O4 (Australian)	“I don’t believe in GPs here, sorry. I haven’t noticed that unless you are going to a medical emergency, I just avoid them.”—A5 (Southern Asian)
Where (location of intervention)	Maternal child health nurse visit		“I think it’s about tapping into those opportunities that already exist, for example, the maternal health appointment is something that all women do.”—O5 (Australian)	“The reason I say maternal child health nurse is because that’s the point of contact.”—A7 (Southern Asian)
	Community-based		“That’s part of access. One thing would be a problem for me if it was far away, I wouldn’t go. Somewhere very local for me is important.”—O4 (Australian)	“I think councils should do a good job in terms of structuring services for families. I really liked having the occasional care, the library, the pool in the same area.”—A9 (Southern Asian)
	Online		“Online is going to be easier for women with babies as it can be a challenge getting places. I guess it’s just finding the time to make that appointment and actually attend.”—O2 (Australian)	“Especially during those time, we had COVID really badly. I think doing the exercise via Zoom was very helpful, because I don’t want to take my baby to crowded places and put the baby at risk.”—A8 (Southern Asian)
Cost and willingness to pay	Free or low cost		“I simply wouldn’t be able to justify the expense whilst on maternity leave.”—O3 (Australian)	“I don’t want to pay high rates to go to a team because I can spend that money on my baby.”—A8 (Southern Asian)
	Depends on needs		“It depends what they offered. If it was just a bit of lifestyle advice I probably wouldn’t. But if it was an exercise class and it had the social aspect as well, then I probably would.”—O4 (Australian)	“If it was the right information, the right service I needed, at the right time, yeah definitely, postpartum I would invest in that.”—A4 (Northeast Asian)
How (delivery mode)	Face-to-face	Theme 4. Building a strong support network for health	“When you’ve got movement, it actually encourages better conversation and often people feel more connected to the conversation because they don’t have to have eye contact.”—O5 (Australian)	“I think it’s better to meet personally than online. I mean, online probably for information, but we still need that social interaction.”—A2 (Northeast Asian)
	Small group		“I like the group setting… Because it was nice to be able to talk to other mums and see different people.”—O4 (Australian)	“Small group would be good. You get to hear what others’ experience, so can learn from them as well.”—A1 (Southeast Asian)
	Peer support		“I think that is a good source of support because we understand what each other is going through and what some of the barriers may be to looking after our health.”—O2 (Australian)	“I would say these classes are amazing. They are mostly operated by ladies. They’re mums and they motivate you with every word they say when you are doing it.”—A5 (Southern Asian)
	Baby-inclusive and family-friendly		“I guess things that would help would be things like exercise classes that are baby-inclusive, things that encompass social connection and community and exercise and health and wellbeing, all kind of wrapped in.”—O8 (New Zealander)	“I think if like family-oriented program, you know, parents and babies and everybody go and do some, uh, programs that would be beneficial.”—A8 (Southern Asian)

GP, general practitioner.

## Data Availability

The data used in the current study are available from the corresponding author on reasonable request.

## Supplementary Materials

The following supporting information can be downloaded at: https://www.mdpi.com/article/10.3390/nu15020472/s1, Supplementary File S1: Online survey; Supplementary File S2: Interview guide; Table S1: Location of residence and ethnicity of survey and interview participants; Table S2: Multivariable regression analysis on survey responses to the preferences for lifestyle intervention after childbirth according to TIDieR framework; Table S3: Sensitivity analysis on survey responses to the preferences for lifestyle intervention after childbirth according to TIDieR framework; Table S4: Survey responses to the preferences for lifestyle intervention after childbirth according to TIDieR framework in Indigenous participants.
