# Supplementary material for: Ethnic Differences in Preferences for Lifestyle Intervention among Women after Childbirth: A Multi-Methods Study in Australia
Source: Nutrients. 2023 Jan 16;15(2):472. doi: 10.3390/nu15020472 (PMC9862492; doi:10.3390/nu15020472)
Supplement: Supplementary file 1 [file nutrients-15-00472-s001.zip › Supplementary materials/Table S1.pdf]

Table S1. Location of residence and ethnicity of survey and interview participants.

	Survey (n=504)	Interview (n=17)
**State or territory**		
Victoria	135 (26.8)	15 (88.2)
New South Wales	157 (31.2)	0 (0.0)
Queensland	104 (20.6)	2 (11.8)
South Australia	30 (6.0)	0 (0.0)
Western Australia	52 (10.3)	0 (0.0)
Australian Capital Territory	10 (2.0)	0 (0.0)
Tasmania	11 (2.2)	0 (0.0)
Northern Territory	5 (1.0)	0 (0.0)
**Ethnicity**		
Oceanian		
Australian	228 (45.2)	7 (41.2)
New Zealander	10 (2.0)	1 (5.9)
Aboriginal and Torres Strait Islander, Maori and Pacific Islander	27 (5.4)	0 (0.0)
Asian		
Northeast Asian	27 (5.4)	3 (17.6)
Southeast Asian	66 (13.1)	1 (5.9)
Southern and Central Asian	81 (16.1)	5 (29.4)
Other		
Northwest European	11 (2.2)	NA
Southern and Eastern European	7 (1.4)	NA
North African and Middle Eastern	9 (1.8)	NA
North American	9 (1.8)	NA
South American	12 (2.4)	NA
Central American	1 (0.2)	NA
Caribbean Islander	1 (0.2)	NA
Central and West African	1 (0.2)	NA
Southern and East African	10 (2.0)	NA
Mixed	4 (0.8)	NA

NA, not applicable. Data are presented as n (%).
