# Supplementary material for: Ethnic Differences in Preferences for Lifestyle Intervention among Women after Childbirth: A Multi-Methods Study in Australia
Source: Nutrients. 2023 Jan 16;15(2):472. doi: 10.3390/nu15020472 (PMC9862492; doi:10.3390/nu15020472)
Supplement: Supplementary file 1 [file nutrients-15-00472-s001.zip › Supplementary materials/Table S2.pdf]

Table S2. Multivariable regression analysis on survey responses to the preferences for lifestyle intervention after childbirth according to TIDieR framework.

**TIDieR elements**	**Model 1**					**Model 2**				
	**Oceanian**	**Asian**		**Other**		**Oceanian**	**Asian**		**Other**	
		**OR (95%CI)**	***P* value**	**OR (95%CI)**	***P* value**		**OR (95%CI)**	***P* value**	**OR (95%CI)**	***P* value**
Intervention content										
Caring for my baby	1.00 (ref)	1.66 (1.06, 2.61)	0.027	0.89 (0.49, 1.59)	0.686	1.00 (ref)	1.51 (0.96, 2.39)	0.077	0.83 (0.46, 1.51)	0.548
Mental health	1.00 (ref)	0.60 (0.36, 0.98)	0.042	1.36 (0.62, 2.98)	0.439	1.00 (ref)	0.62 (0.37, 1.03)	0.064	1.40 (0.64, 3.07)	0.406
Send me reminders and prompts	1.00 (ref)	1.47 (0.95, 2.28)	0.086	0.64 (0.36, 1.15)	0.137	1.00 (ref)	1.49 (0.95, 2.32)	0.081	0.65 (0.36, 1.16)	0.143
Intervention provider										
Someone with expertise in women's health, e.g. health professional	1.00 (ref)	0.61 (0.29, 1.27)	0.186	4.67 (0.60, 36.31)	0.140	1.00 (ref)	0.63 (0.30, 1.34)	0.230	4.81 (0.62, 37.48)	0.134
Intervention commencement time [a]										
6 months or earlier	1.00 (ref)	0.15 (0.04, 0.48)	0.002	0.47 (0.08, 2.70)	0.394	1.00 (ref)	0.16 (0.05, 0.55)	0.004	0.51 (0.09, 3.00)	0.456
Intervention duration [b]										
6 months or longer	1.00 (ref)	0.48 (0.30, 0.76)	0.002	0.84 (0.42, 1.65)	0.604	1.00 (ref)	0.50 (0.31, 0.81)	0.005	0.87 (0.44, 1.72)	0.681
Intervention frequency [c]										
Monthly, fortnightly or weekly	1.00 (ref)	0.47 (0.29, 0.78)	0.003	0.90 (0.43, 1.88)	0.785	1.00 (ref)	0.46 (0.28, 0.76)	0.002	0.88 (0.42, 1.84)	0.739
Duration of session [d]										
More than 30 minutes	1.00 (ref)	0.55 (0.36, 0.84)	0.006	0.84 (0.47, 1.49)	0.549	1.00 (ref)	0.56 (0.36, 0.86)	0.008	0.85 (0.48, 1.51)	0.574
Delivery mode										
Individual video or phone consultation	1.00 (ref)	1.61 (1.05, 2.48)	0.029	0.86 (0.47, 1.58)	0.628	1.00 (ref)	1.59 (1.03, 2.45)	0.037	0.85 (0.47, 1.56)	0.609
Group video consultation	1.00 (ref)	1.98 (1.20, 3.25)	0.007	0.86 (0.41, 1.82)	0.696	1.00 (ref)	1.95 (1.18, 3.21)	0.010	0.86 (0.40, 1.81)	0.682

OR, odds ratio; CI, confidence interval.

Model 1 adjusted for age, education, employment and household income; Model 2 adjusted for age, education, employment, household income and number of children.

For all outcome variables: 1=selected, 0=not selected, otherwise indicated.

[a] 1=6 months or earlier, 0=after 6 months.

[b] 1=6 months or longer, 0=3 months or shorter.

[c] 1= monthly, fortnightly or weekly, 0=every 3 to 6 months.

[d] 1=more than 30 minutes, 0=less than 30 minutes.
