# Supplementary material for: Ethnic Differences in Preferences for Lifestyle Intervention among Women after Childbirth: A Multi-Methods Study in Australia
Source: Nutrients. 2023 Jan 16;15(2):472. doi: 10.3390/nu15020472 (PMC9862492; doi:10.3390/nu15020472)
Supplement: Supplementary file 1 [file nutrients-15-00472-s001.zip › Supplementary materials/Table S3.pdf]

Table S3. Sensitivity analysis on survey responses to the preferences for lifestyle intervention after childbirth according to TIDieR framework.

**TIDieR elements**	**Oceanian (n=238)** [*]	**Asian (n=174)**	**Other (n=65)**	***P* value**
Interest in lifestyle intervention				
Yes	216 (90.8)	163 (93.7)	56 (86.2)	0.178
Intervention content				
Knowledge				
Women's health	198 (83.2)	148 (85.1)	55 (84.6)	0.870
Breastfeeding	165 (69.3)	115 (66.1)	45 (69.2)	0.769
Caring for my baby	137 (57.6)	124 (71.3)	37 (56.9)	0.011 [a]
Children's health	149 (62.6)	124 (71.3)	42 (64.6)	0.180
Mum's diet	165 (69.3)	108 (62.1)	38 (58.5)	0.147
Exercise after birth	177 (74.4)	118 (67.8)	46 (70.8)	0.344
Weight management	167 (70.2)	132 (75.9)	42 (64.6)	0.188
Mental health	190 (79.8)	120 (69.0)	53 (81.5)	0.021 [b]
Preventing diabetes or heart disease	69 (29.0)	67 (38.5)	17 (26.2)	0.068
Skills and strategy				
How to determine the credibility of health information	66 (27.7)	43 (24.7)	20 (30.8)	0.609
How to set goals and action plans for health	116 (48.7)	75 (43.1)	25 (38.5)	0.259
How to set aside time for health	120 (50.4)	83 (47.7)	35 (53.9)	0.681
Self-recording diet or physical activity	63 (26.5)	56 (32.2)	13 (20.0)	0.146
Monitoring blood tests and other health outcomes	55 (23.1)	45 (25.9)	18 (27.7)	0.683
Someone to monitor my progress	155 (65.1)	101 (58.1)	43 (66.2)	0.281
Send me reminders and prompts	131 (55.0)	110 (63.2)	29 (44.6)	0.028 [c]
Social support for health	163 (68.5)	109 (62.6)	43 (66.2)	0.465
Questions to ask my doctor	100 (42.0)	76 (43.7)	23 (35.4)	0.508
Intervention provider				
Someone with expertise in women's health, e.g. health professional	217 (91.2)	153 (87.9)	64 (98.5)	0.040 [d]
Someone with expertise in children's health, e.g. health professional	124 (52.1)	100 (57.5)	34 (52.3)	0.532
Another mum	63 (26.5)	45 (25.9)	15 (23.1)	0.857
Intervention commencement time				0.003 [e]
6 weeks or earlier	81 (34.5)	52 (30.1)	24 (37.5)	
7 weeks to 3 months	101 (43.0)	65 (37.6)	31 (48.4)	
4 to 6 months	49 (20.9)	39 (22.5)	6 (9.4)	
After 6 months	4 (1.7)	17 (9.8)	3 (4.7)	
Intervention duration				0.025 [f]
≤ 1 month	21 (9.0)	34 (19.7)	9 (13.9)	
3 months	31 (13.3)	29 (16.8)	9 (13.9)	
6 months	74 (31.8)	36 (20.8)	15 (23.1)	
1 year	107 (45.9)	74 (42.8)	32 (49.2)	
Intervention frequency				<0.001 [g,h]
Every 6 months	13 (5.6)	11 (6.5)	3 (4.7)	

Every 3 months	32 (13.8)	46 (27.1)	10 (15.6)	
Every month	84 (36.2)	76 (44.7)	17 (26.6)	
Every fortnight	60 (25.9)	29 (17.1)	21 (32.8)	
Every week	43 (18.5)	8 (4.7)	13 (20.3)	
Duration of session				0.030 [i]
Less than 15 minutes	13 (5.5)	10 (5.8)	5 (7.7)	
Between 15 to 30 minutes	88 (37.0)	94 (54.3)	27 (41.5)	
Between 30 to 45 minutes	100 (42.0)	48 (27.8)	23 (35.4)	
More than 45 minutes	37 (15.6)	21 (12.1)	10 (15.4)	
Delivery mode				
Individual video or phone consultation	94 (39.5)	86 (49.4)	22 (33.9)	0.043 [j]
Individual face-to-face consultation	140 (58.8)	86 (49.4)	40 (61.5)	0.100
Group video consultation	49 (20.6)	52 (29.9)	11 (16.9)	0.036 [k]
Group face-to-face consultation	89 (37.4)	64 (36.8)	28 (43.1)	0.651
Location of intervention				
Online	170 (71.4)	115 (66.1)	43 (66.2)	0.456
Maternal child health nurse visit	180 (75.6)	139 (79.9)	48 (73.9)	0.489
GP clinic	121 (50.8)	92 (52.9)	37 (56.9)	0.677
Mother's group or playgroup	122 (51.3)	103 (59.2)	35 (53.9)	0.277

GP, general practitioner. Data are presented as n (%).

[*] Indigenous Peoples including Australian Aboriginal, Torres Strait Islanders, Maori and Pacific Islanders were excluded (n=27) from the Oceanian group.

[a] Significant pairwise differences between Oceanian and Asian (Bonferroni-adjusted *P*=0.013).

[b] Significant pairwise differences between Oceanian and Asian (Bonferroni-adjusted *P*=0.035).

[c] Significant pairwise differences between Asian and Other (Bonferroni-adjusted *P*=0.028).

[d] Significant pairwise differences between Asian and Other (Bonferroni-adjusted *P*=0.037).

[e] Significant pairwise differences between Oceanian and Asian (Bonferroni-adjusted *P*=0.008).

[f] Significant pairwise differences between Oceanian and Asian (Bonferroni-adjusted *P*=0.010).

[g] Significant pairwise differences between Oceanian and Asian (Bonferroni-adjusted *P*<0.001).

[h] Significant pairwise differences between Asian and Other (Bonferroni-adjusted *P*<0.001).

[i] Significant pairwise differences between Oceanian and Asian (Bonferroni-adjusted *P*=0.012).

[j] No significant pairwise differences found after Bonferroni correction.

[k] No significant pairwise differences found after Bonferroni correction.
