# Supplementary material for: Ethnic Differences in Preferences for Lifestyle Intervention among Women after Childbirth: A Multi-Methods Study in Australia
Source: Nutrients. 2023 Jan 16;15(2):472. doi: 10.3390/nu15020472 (PMC9862492; doi:10.3390/nu15020472)
Supplement: Supplementary file 1 [file nutrients-15-00472-s001.zip › Supplementary materials/Table S4.pdf]

Table S4. Survey responses to the preferences for lifestyle intervention after childbirth according to TIDieR framework in Indigenous participants (n=27).

**TIDieR elements**	**n**	**%**
Interest in lifestyle intervention		
Yes	23	85.2
Intervention content		
Knowledge		
Women's health	24	88.9
Breastfeeding	19	70.4
Caring for my baby	21	77.8
Children's health	21	77.8
Mum's diet	20	74.1
Exercise after birth	21	77.8
Weight management	20	74.1
Mental health	26	96.3
Preventing diabetes or heart disease	13	48.2
Skills and strategy		
How to determine the credibility of health information	12	44.4
How to set goals and action plans for health	15	55.6
How to set aside time for health	16	59.3
Self-recording diet or physical activity	10	37.0
Monitoring blood tests and other health outcomes	10	37.0
Someone to monitor my progress	21	77.8
Send me reminders and prompts	20	74.1
Social support for health	17	63.0
Questions to ask my doctor	14	51.9
Intervention provider		
Someone with expertise in women's health, e.g. health professional	26	96.3
Someone with expertise in children's health, e.g. health professional	18	66.7
Another mum	9	33.3
Intervention commencement time		
6 weeks or earlier	12	46.2
7 weeks to 3 months	11	42.3
4 to 6 months	3	11.5
After 6 months	0	0.0
Intervention duration		
≤ 1 month	1	3.7
3 months	3	11.1
6 months	6	22.2
1 year	17	63.0
Intervention frequency		
Every 6 months	0	0.0
Every 3 months	3	11.1

Every month	10	37.0
Every fortnight	8	29.6
Every week	6	22.2
Duration of session		
Less than 15 minutes	2	7.4
Between 15 to 30 minutes	9	33.3
Between 30 to 45 minutes	10	37.0
More than 45 minutes	6	22.2
Delivery mode		
Individual video or phone consultation	7	25.9
Individual face-to-face consultation	14	51.9
Group video consultation	3	11.1
Group face-to-face consultation	9	33.3
Location of intervention		
Online	18	66.7
Maternal child health nurse visit	16	59.3
GP clinic	13	48.2
Mother's group or playgroup	12	44.4

GP, general practitioner.

Indigenous participants included Australian Aboriginal, Torres Strait Islanders, Maori and Pacific Islanders.
